# Tolerance of Olaparib in a Patient With Unresectable Serous Gynecologic Cancer and End-Stage Renal Disease

**DOI:** 10.7759/cureus.36505

**Published:** 2023-03-22

**Authors:** Kelsey C Goon, Jill Alldredge

**Affiliations:** 1 Obstetrics and Gynecology, University of Colorado Hospital, Aurora, USA; 2 Obstetrics and Gynecology/Gynecologic Oncology, University of Colorado Hospital, Aurora, USA

**Keywords:** serous carcinoma, dialysis, end stage renal disease (esrd), advanced ovarian cancer, parp-inhibitors

## Abstract

Poly (adenosine diphosphate-ribose) polymerase enzyme (PARP) inhibitors have risen in popularity for the treatment of gynecologic cancers, largely due to an expansion of applications with the discovery of more genetic mutations that manifest as homologous recombination deficiency. PARP inhibitors further represent an appealing management option as oral maintenance or monotherapy. While dose adjustments exist for mild kidney dysfunction, little is published about the use of PARP inhibitors in patients with severe renal dysfunction. We present a case of advanced, serous gynecologic cancer in a patient who was ineligible for surgery due to cardiac and renal comorbidities and treated with olaparib for nine months without direct adverse effects, despite a paucity of literature supporting the use or dosing of olaparib in patients requiring dialysis. Further studies are needed to better establish the safety, efficacy, and appropriate dose modification for patients with end-stage renal disease.

## Introduction

Poly (adenosine diphosphate-ribose) polymerase enzyme (PARP) inhibitors have seen expanded use in the realm of gynecologic oncology. This class of therapy acts by inhibiting deoxyribonucleic acid (DNA) repair and apoptosis [1]. As such, PARP inhibitors are approved by the Food and Drug Administration (FDA) for use as maintenance therapy in ovarian cancer with germline or somatic BRCA gene mutations and recurrent platinum-sensitive disease, in addition to several indications in the treatment of breast, pancreatic, and prostate cancer [1-3]. While manufacturers include recommended dose modifications for mild and moderate renal impairment, little is published regarding the safety or recommended dose adjustments for PARP inhibitor use in patients with severe renal dysfunction. Olaparib is specifically recommended for a starting dose of 300 mg twice daily, with a reduction to 200 mg twice daily for patients with moderate renal impairment, defined as creatinine clearance between 31-50 mL/min. The pharmacokinetics of olaparib in renal impairment with a creatinine clearance of less than 30 mL/min have explicitly not been evaluated [3].

The goal of this report is to share an example of olaparib usage in a patient with advanced gynecologic cancer and a germline BRCA mutation complicated by medical comorbidities that precluded surgical management, including end-stage renal disease (ESRD) requiring hemodialysis.

## Case presentation

A 70-year-old woman with a medical history notable for ESRD on hemodialysis, chronic systolic heart failure, anemia, atrial fibrillation with a rapid ventricular response, breast cancer in remission, coronary artery disease, hyperlipidemia, hypertension, hypothyroidism, and systemic lupus erythematosus was found to have a complex pelvic mass and possible periaortic lymphadenopathy during admission for exacerbation of heart failure with pulmonary edema and a spontaneous retroperitoneal hemorrhage attributed to chronic anticoagulation. Her history was further notable for four coronary artery bypass graft procedures and a renal transplant.

**Figure 1:**
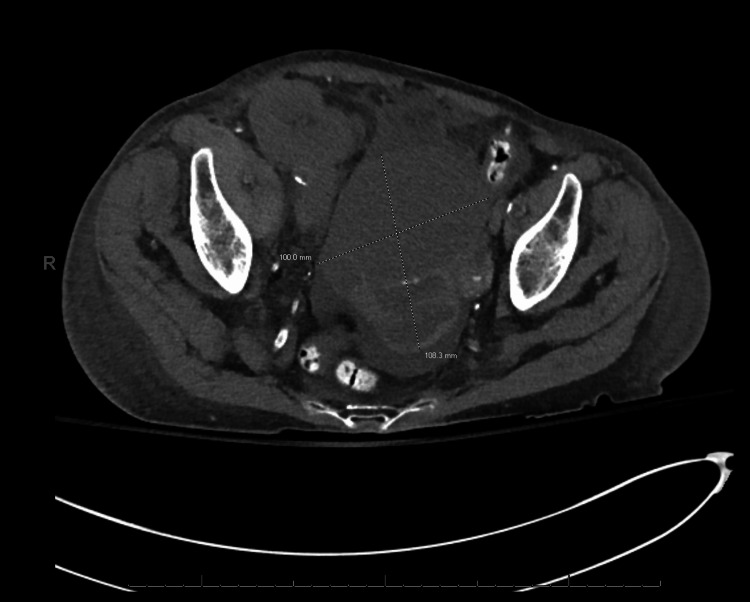
Transverse view of complex pelvic mass

**Figure 2:**
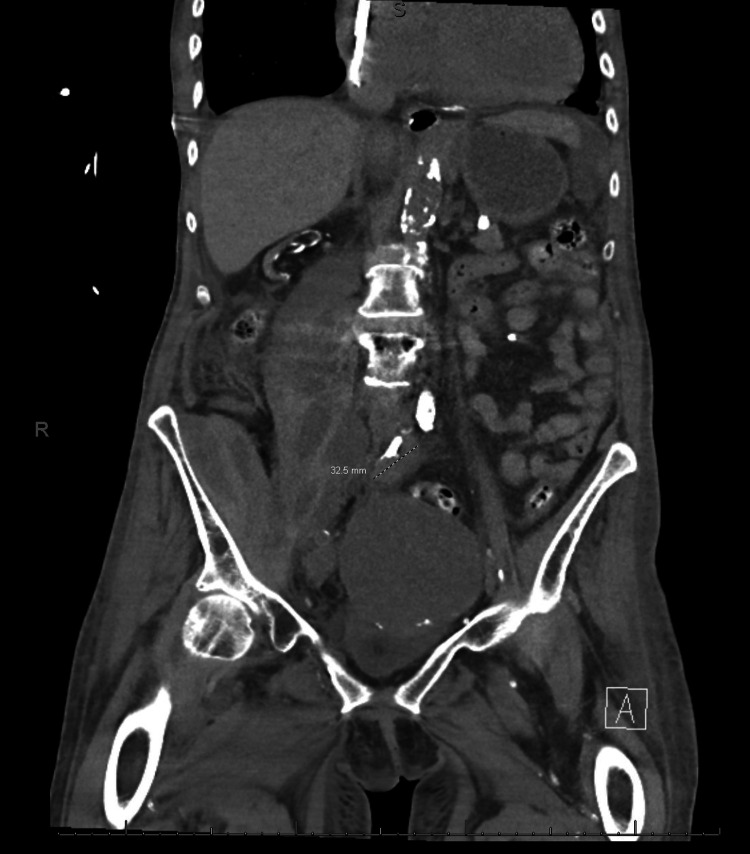
Coronal view of complex pelvic mass with suspicious periaortic lesion

**Figure 3:**
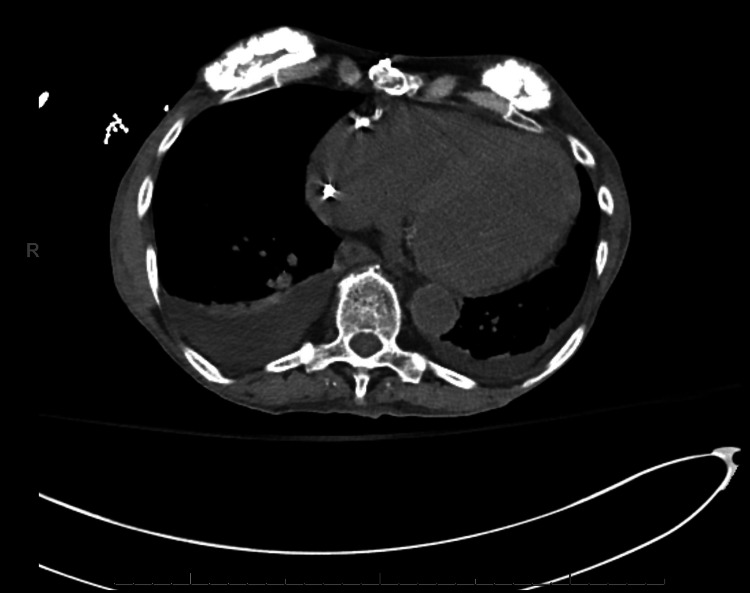
Transverse view of bilateral pleural effusions

Serum tumor markers were notable for an elevated CA125, normal CA19-9, and normal CEA. Ultrasound-guided biopsy of the pelvic mass was performed by the Interventional Radiology service under conscious sedation and local anesthesia. The biopsy demonstrated malignant cells with positive immunohistochemical staining for CK7, PAX8, estrogen receptor, WT-1, and p16, and negative staining for CK20, CDX2, and progesterone receptor. This was consistent with adenocarcinoma and suggestive of a serous gynecologic primary malignancy. Given the patient's history of breast cancer and this new gynecologic malignancy, she was recommended and accepted a referral to genetic counseling. Based on her counseling, she elected for germline panel testing and was found to have a germline BRCA-1 mutation. Due to her multiple comorbidities, the interpretation of her chest imaging which included bilateral pleural effusions and a suspicious periaortic lesion was inconclusive between stage IIIC and stage IVB. Figures 1-3 demonstrate the patient's imaging at the time of diagnosis, noting limitations in image quality secondary to the inability to utilize intravenous contrast due to her renal function. 

She was counseled regarding management options ranging between disease-directed treatment with chemotherapy alone with palliative intention, and strictly comfort-care with hospice. After counseling, she strongly desired to pursue disease-directed therapy. She was initially managed with single-agent carboplatin at 150 mg set dose for three cycles, with hemodialysis scheduled 24 hours after each cycle. After three cycles, imaging demonstrated stable disease, and she ultimately underwent six cycles of carboplatin. Her initial treatment course was notable for several one-week delays for thrombocytopenia. A multidisciplinary discussion with the patient’s cardiac and renal teams concluded that surgical management would be absolutely contraindicated for this patient. At this juncture, she continued to express a strong desire for disease-directed therapy. Thus, she transitioned to maintenance therapy.

Given her BRCA mutation, PARP inhibitors were the first to be considered for maintenance monotherapy. The patient was counseled regarding the absence of published safety data in patients with ESRD and through shared decision-making elected to proceed with olaparib monotherapy. She received four weeks of olaparib dosed at 100 mg twice daily, which was then up-titrated to 200 mg twice daily. After four weeks of the increased dosing, she experienced nausea and poor appetite albeit in the setting of pyelitis, at which point her dosage was decreased back to 100 mg twice daily. Imaging at six months of maintenance therapy demonstrated improvement in size of the pelvic mass. Olaparib dosing was up-titrated to 100 mg in the morning and 200 mg in the evening, which the patient tolerated without adverse effects. At nine months of therapy, imaging demonstrated progression of the disease and she was transitioned to a new line of therapy with gemcitabine and bevacizumab which she continued for nine months until she decompensated from her cardiac disease and passed. Outside of the episode of pyelitis, she did not experience any treatment-related adverse effects, changes to her quality of life, or ability to perform activities of daily living during her treatment with olaparib.

## Discussion

Patients with complex comorbidities present management challenges as they can have contraindications to standards of care. Surgical management was directly contraindicated for the patient in this case, and medical management with PARP inhibitors did not have discrete recommendations for her profoundly impaired renal function. The tolerance of dose-reduced olaparib for nine months in this patient with ESRD without adverse impact on her quality of life represents a potential model of care in hemodialysis-requiring patients with BRCA mutations.

Similar to other PARP inhibitors, the classic toxicities of olaparib include nausea, vomiting, cytopenias, fatigue, and dysgeusia [1-3]. Of these, cytopenias represent the most common reason for therapy reduction or interruption [1-3]. Compared to niraparib and rucaparib, olaparib less frequently requires adjustment in therapy, which makes it a more appealing option in patients with impaired excretion [2]. Olaparib excretion is partially renal and partially hepatic [3-4]. The standard dosing of olaparib is 300 mg twice daily for a total daily dose of 600 mg. For adverse reactions, the recommended dose reduction is by 50 mg per dose, down to a total daily dose of 400 mg [3]. For co-administration with a CYP3A inhibitor, a dose reduction to a total daily dose of 200 mg or 300mg, depending on the strength of inhibition, is recommended [3].

Table 1, which is adapted from the Lynparza (olaparib) FDA-approved package insert, demonstrates the recommended dose adjustments by renal function [3]. For patients with renal impairment, no dose adjustments are suggested for mild impairment (creatinine clearance 51-80 mL/min), and a reduction to a total daily dose of 400 mg is recommended for patients with moderate dysfunction (creatinine clearance 31-50 mL/min) [2]. Explicitly, the pharmacokinetics of olaparib or any other PARP inhibitor have not been evaluated in patients with severe renal dysfunction or use of hemodialysis.

**Table 1:** Recommended dose adjustments for olaparib by renal function Adapted from the Lynparza (olaparib) FDA-approved package insert [3].

Degree of Renal Impairment	Creatinine Clearance (mL/min)	Increase in Mean Exposure	Recommended Dosage
None	> 80 mL/min	-	300 mg, twice daily
Mild	51-80 mL/min	24%	300 mg, twice daily
Moderate	31-50 mL/min	44%	200 mg, twice daily
Severe	≤ 30 mL/min	No data	No data

Furthermore, the relationship between PARP inhibitors and the kidneys has proven complex and remains an ongoing area of investigation. On one side, recent animal models have suggested a nephroprotective role of PARP inhibitors in the treatment of ischemic acute kidney injury. In this scenario, damage from reactive oxygen species is proposed to lead to the overactivation of PARP, which in turn results in renal tubular necrosis. Animal models have suggested that PARP inhibitors in this acute setting may represent an opportunity to decrease necrotic cell injury [5-6]. On the other hand, in vitro studies have suggested that PARP inhibitors impair several renal transporters in the proximal tubule including multidrug and toxin extruders (MATE) and the organic cation transporters (OCT) resulting in a drug-induced elevation in serum creatinine. This elevation in serum creatinine is noted to stabilize over time and normalize, if not necessarily return to baseline, after discontinuation [5,7].

Ultimately, the concern with the use of PARP inhibitors in patients with renal disease remains the accumulation of serum drug concentrations and the subsequent possibility of an increased rate of toxicity. On our literature review, there is currently a single case report investigating the prolonged use of rucaparib in a patient with a BRCA mutation and concurrent breast and ovarian cancer, on three-times-a-week hemodialysis. That study was funded by Clovis Oncology for the assessment of peak and trough values of rucaparib, and demonstrated non-toxic concentrations in the setting of hemodialysis usage at a 66% dose reduction [8]. When our case is viewed alongside this single-patient assessment of rucaparib usage in a patient requiring hemodialysis, it supports the cautious and well-supervised usage of PARP inhibitors in patients with severe renal impairment.

## Conclusions

To our knowledge, this is the first report to demonstrate the safe usage of olaparib in a patient with ESRD requiring hemodialysis. We present a potential path forward for disease-directed management of ovarian cancer in patients with advanced renal disease, in whom treatment options are currently limited. Additional investigation into the pharmacokinetics of PARP inhibitors in the setting of hemodialysis is warranted. Studies examining the serum level of olaparib in patients undergoing hemodialysis would add to the current understanding of safe dosing and titration.
